# Japanese Physicians’ Views on Drug Post-Marketing Surveillance

**DOI:** 10.14740/jocmr2328w

**Published:** 2015-10-23

**Authors:** Kazuki Maeda, Rumi Katashima, Keisuke Ishizawa, Hiroaki Yanagawa

**Affiliations:** a Clinical Trial Center for Developmental Therapeutics, Tokushima University Hospital, Tokushima, Japan; b Institutional Review Board, Tokushima University Hospital, Tokushima, Japan

**Keywords:** Post-marketing surveillance, Physician, View, Japan, Research ethics, Infrastructure

## Abstract

**Background:**

Registration trials leading to the approval of drugs are paramount in drug development. After approval, continuous efforts are necessary to ensure proper use of the approved drugs. In Japan, post-marketing surveillance (PMS) by drug companies is conducted in accordance with good post-marketing study practice (GPSP). Although the global standard for pharmacovigilance is incorporated into GPSP, attention has recently been focused on disassociating them. In this study, we examined physicians’ views on PMS with the aim of conducting PMS more effectively.

**Methods:**

We retrospectively reviewed records between 2009 and 2013 from the institutional review board of Tokushima University Hospital, an academic hospital in rural Japan. The annual number of times PMS was performed was then determined. Next, we assessed physicians’ attitudes toward drug PMS, including ethical issues, in a cross-sectional study using a questionnaire designed for this study. Five- and two-point scales were used. The questionnaire was distributed in 2014 to 221 physicians listed as investigators in PMS contracts.

**Results:**

Of the 221 physicians, 103 (46.6%) responded to the questionnaire. About 50% of the respondents had experience writing PMS reports. Many of the physicians considered PMS to be important but burdensome. Furthermore, from the viewpoint of research ethics, many physicians considered it improper within the present PMS framework to collect and provide data beyond the scope of routine clinical practice without obtaining informed consent in the case of extra blood sampling, provision of images, monitoring and controlled studies.

**Conclusions:**

Beyond practical factors such as workload, attention should be given to establishing an ethical infrastructure and globally harmonized system with regard to the Japanese PMS system. Given the limitations of this single-institution study, further research is needed to collect information for developing a suitable infrastructure.

## Introduction

Registration trials leading to the approval of drugs are paramount in drug development. In Japan, the Ministry of Health, Labour and Welfare (MHLW) is responsible for approval, while the Pharmaceuticals and Medical Device Agency (PMDA) is responsible for the operational aspects of drug development. After approval, continuous efforts are necessary to ensure proper use of the approved drugs. To expand the safety profile, various systems are currently in place in Japan. For example, health care professionals are required to provide a report to the MHLW if they detect any disorders thought to be caused by adverse drug reactions. This system was started on a voluntary basis in 1967, and then stipulated in the Pharmaceutical Affairs Law in 2003. Another is the post-marketing surveillance (PMS) system initiated by the drug companies themselves. Japanese drug companies must make provisions for the conduct of PMS in accordance with good post-marketing study practice (GPSP) [1]. In 2004, the E2E guideline was introduced as a global standard for pharmacovigilance [2], and since its introduction into Japan in 2005, the concept has been incorporated into GPSP. Another new concept of pharmacovigilance is the risk management plan (RMP) [3]. Since its introduction in Japan in 2012 [4], various attempts have been made to modify the design of PMS to make it suitable for RMPs. In line with this, increased attention has been paid to the roles of physicians in RMPs, such as contributing to PMS design, collecting data for safety profiles, and better explaining risks to patients.

A dramatic change in Japanese registration trials occurred with the increase of global trials. Previously, delays in approval of new drugs were a problem in Japan compared with the United States and other countries [5]. Recent reports of a gradual decrease in drug lag suggest several important contributing factors, for example, the inclusion of Japanese subjects in global clinical trials and improved early trial design and planning [6, 7]. In parallel, an increasing number of drugs have been approved with less data from Japanese subjects, and there has been an increase in data collection for efficacy profiles within the PMS framework; the latter was originally conducted to collect data for safety profiles only [8].

In this study, with the aim of conducting PMS more effectively, we focused on physicians’ roles in collecting data during the post-marketing phase. We examined physicians’ views on PMS via a questionnaire survey, and herein present the results of our analysis.

## Methods

In Tokushima University Hospital, approval by the institutional review board (IRB) is mandatory prior to conducting PMS. First, we retrospectively reviewed records from the Tokushima University Hospital IRB for the period 2009 - 2013, and determined the annual number of times PMS was performed.

Next, we assessed physicians’ attitudes toward drug PMS, including ethical issues, in a cross-sectional study conducted at Tokushima University Hospital. A questionnaire was designed for this study. The questionnaire was anonymous and consisted of 17 questions spread across three sections. The first consisted of four demographic questions, including experience of providing voluntary reports on adverse drug reactions. The second consisted of seven questions aimed at determining the physicians’ experience of and attitude toward PMS. The third and final sections consisted of six questions concerning ethical issues in PMS. Responses to section 2 were scored on a five-point Likert scale (1 = strongly disagree, 2 = disagree, 3 = neutral, 4 = agree, 5 = strongly agree). In the third section, a two-point scale (yes/no) was used.

The questionnaire was distributed in October 2014 to 221 physicians who were listed as investigators in the PMS contracts reviewed by the IRB in 2012 and 2013. All completed questionnaires were collected anonymously.

Data were expressed as the mean ± SD of scores or n (%). We compared the views of physicians based on their experiences of voluntary reporting adverse drug reactions, and analyzed the differences using the *t*-test. P-values < 0.05 were considered significant. All P-values were based on two-sided tests. All statistical analyses were carried out using SPSS software, version 21.0 (IBM SPSS Statistics Base Authorized). This study was approved by the Ethics Committee of Tokushima University Hospital.

## Results

### Number of PMS studies for drugs and medical devices

Table 1 shows the number of times that PMS for drugs and medical devices was conducted with approval of the IRB at Tokushima University Hospital each year during the 5-year study period. About 90% of these were for drugs.

**Table 1:** Annual and Total Number of Times Post-Marketing Surveillance (PMS) of Drugs and Medical Devices Was Conducted in Tokushima University Hospital During the Study Period

	PMS		
	Drug	Medical device	Total
2009	62	3	65
2010	49	5	54
2011	51	4	55
2012	38	3	41
2013	47	8	55
Total	247	23	270

### Questionnaire survey and respondent characteristics

Of the 221 physicians surveyed at Tokushima University Hospital, 103 (46.6%) completed the questionnaire and were included in the analysis. Respondents included 83 men (81.4%) and 19 women (18.6%); one (1.0%) respondent provided no response for gender. The age distribution was as follows: 24 - 29 years (n = 1, 1.0%), 30 - 39 years (n = 42, 40.8%), 40 - 49 years (n = 42, 40.8%), and ≥ 50 years (n = 18, 17.5%). Length of experience as a physician was as follows: 1 - 5 years (n = 1, 1.0%), 6 - 10 years (n = 17, 16.8%), 11 - 15 years (n = 32, 31.7%), ≥ 16 years (n = 51, 50.5%), and no response (n = 2, 1.9%). Experience of voluntary reporting of adverse drug reactions was as follows: yes (n = 33, 32.0%) and no (n = 70, 68.0%).

### Physicians’ experience and views of PMS

Respondents were asked about the number of PMS reports completed, electronic data capturing (EDC) responses, and responses to queries. As shown in Table 2, about 50% of respondents had experience writing PMS reports, and of these, about 50% had experience with EDC reports. Experience responding to queries was noted by about 40% of respondents.

**Table 2:** Number of PMS Reports, Electronic Data Capturing Responses, and Responses to Queries

	0	1 - 5	6 - 10	11 - 15	> 16	No response
PMS reports	48 (46.6%)	40 (38.8%)	5 (4.9%)	0	9 (8.7%)	1 (1.0%)
Electronic data capturing responses	77 (74.7%)	17 (16.5%)	2 (1.9%)	1 (1.0%)	5 (4.9%)	1 (1.0%)
Responses to queries	60 (58.2%)	34 (33.0%)	5 (4.9%)	1 (1.0%)	2 (1.9%)	1 (1.0%)

Respondents’ views on PMS are shown in Table 3. Less than 10% of respondents provided negative answers (disagree or strongly disagree) concerning the importance of PMS. As for the workload, no respondents provided negative answers but no specific tendency was observed in their views on change in workload. Negative answers (disagree or strongly disagree) were more prevalent than positive answers (agree or strongly agree) regarding feedback of PMS results.

**Table 3:** Respondents’ Views on the Importance of PMS and the Associated Workload as Well as Feedback of PMS Results to Physicians

	Strongly disagree (score 1)	Disagree (score 2)	Neutral (score 3)	Agree (score 4)	Strongly agree (score 5)	No response
PMS is important	2 (1.9%)	4 (3.9%)	34 (33.0%)	32 (31.1%)	31 (30.1%)	0
The workload of PMS is a burden	0	0	10 (9.7%)	29 (28.2%)	26 (25.2%)	38 (36.9%)
The workload of PMS has been increasing	17 (16.5%)	3 (2.9%)	17 (16.5%)	15 (14.6%)	12 (11.7%)	39 (37.9%)
Feedback of PMS results to physicians is satisfactory	14 (13.6%)	14 (13.6%)	27 (26.2%)	10 (9.7%)	9 (8.7%)	29 (28.2%)

### Relationship between experience of voluntary reporting and attitudes toward PMS

Since the above attitude of physicians toward PMS may influence their views, we compared mean scores of views between respondents with experience of voluntary reporting of adverse drug reactions and those without. There was no demographic difference between the two groups (data not shown). As shown in Table 4, respondents with experience showed significantly higher scores for the question pertaining to workload (“The workload with PMS is a burden”); however, no significant difference was observed regarding “PMS is important” or “Workload of PMS has been increasing”.

**Table 4:** Relationship Between Experience of Voluntary Reporting and Views on PMS

	Mean with SDs of scores		
	Voluntary reporting experience	No experience	P
PMS is important	4.09 ± 0.91 (n = 33)	3.71 ± 0.98 (n = 70)	0.07
The workload of PMS is a burden	4.46 ± 0.71 (n = 26)	4.10 ± 0.68 (n = 39)	0.04
The workload of PMS has been increasing	3.16 ± 1.70 (n = 25)	2.95 ± 1.30 (n = 39)	0.58

### Physicians’ views on the ethical issues of PMS

In Japan, PMS is conducted in accordance with regulations; essentially, data from clinical practice are required but ethics review is not mandatory. Recently, several PMS studies were conducted to collect data beyond the scope of routine clinical practice. After a brief explanation, we asked the physicians how they would feel about having to do so. For this purpose, we first presented three frameworks and asked the physicians to select the one that they considered proper to collect and provide data beyond the scope of routine clinical practice. In the remaining five questions, we asked whether they deemed it acceptable to collect and provide data without informed consent for the following five items: extra blood sampling, questionnaire survey, provision of images, monitoring and controlled studies, respectively.

**Figure 1:**
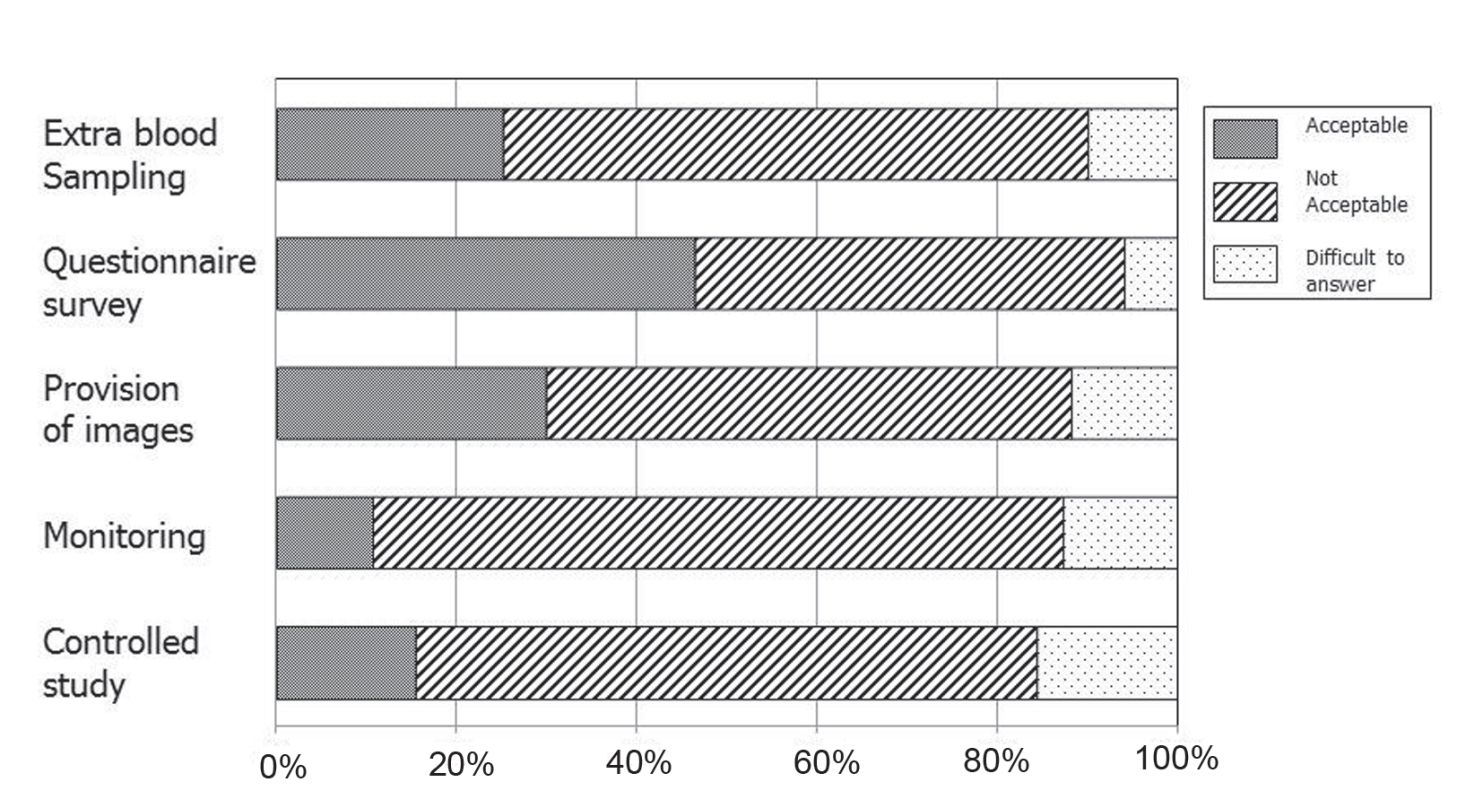
Respondents’ views on the acceptability to collect and provide data in various issues without obtaining informed consent in the current framework of post-marketing surveillance.

A total of 37 respondents (35.9%) selected “collect and provide data in the framework of PMS without informed consent”, 44 (42.7%) selected “collect and provide data beyond routine clinical practice, separate from PMS with informed consent”, 20 (19.4%) selected “conduct a clinical study separate from PMS and collect and provide data with informed consent”, and two (1.9%) provided no response. As shown in Figure 1, the use of questionnaire surveys was accepted by almost half of the respondents, whereas other procedures were supported by 30% or less of the respondents.

## Discussion

Regarding pharmacovigilance, which is an important step in drug use, we focused on physicians’ roles in collecting data during the post-marketing phase, and by a questionnaire survey, we examined their views on practical issues in PMS. As expected, less than 10% of respondents provided negative answers (disagree or strongly disagree) regarding the importance of PMS, and the workload of PMS (Table 3). On the other hand, no specific tendency was observed in their views on the change in workload. PMS workload may vary among physicians depending, for example, on the type of drug and method of data collection, and as a result, the findings likely reflected this variety. Future research is, however, required to examine this further.

Since the introduction of Good Clinical Practice in 1997, the infrastructure for registration trials in Japan has improved. The contribution of clinical trial support staff such as clinical research coordinators and administrative officers is now widely recognized for practical pursuits and quality assurance during registration trials. Although clinical research coordinators support of registration trials at clinics is usually via a site management organization, those working in key hospitals are usually employed by the institution and belong to the clinical trial division of the department of pharmacy. Application of a similar infrastructure might therefore be a good strategy for the practical pursuit and quality assurance of PMS, as shown previously [9]. Nevertheless, such practices are still being performed on a preliminary basis only. In a survey in Tokyo, Watanabe et al revealed that pharmacy departments in 47% of hospitals examined were contributing to post-marketing surveillance; however, the contribution was mainly in administrative matters, not in the reports themselves [10]. As shown in Table 4, respondents with experience of voluntary reporting of adverse drug reactions showed significantly higher scores for the item “Workload of PMS is a burden”. The results suggest that physicians who are motivated to participate in pharmacovigilance have a realistic attitude toward PMS. The establishment of an infrastructure to support such activities is therefore warranted for collection of high-quality data. In addition, considering the fact that few physicians indicated satisfaction with the feedback of PMS results (Table 3), emphasis on the significance of PMS, with appropriate feedback of PMS results, may lead to physicians contributing more to pharmacovigilance. Vallano et al [11] established regular educational pharmacovigilance sessions in a teaching hospital, and reported that most physicians who attended were satisfied and considered them useful. Educational sessions including feedback of PMS results could therefore be an important strategy.

A remaining issue in Japanese PMS is harmonization with global standards. In countries where the concept of the International Conference on Harmonization of Technical Requirements for Registration of Pharmaceuticals for Human Use is accepted, pharmacovigilance is performed in line with E2E, and sponsors follow good epidemiological practices for observational studies as well as internationally accepted guidelines, such as those endorsed by the International Society for Pharmacoepidemiology [2]. On the other hand, Japanese regulations, including GPSP, are applied to PMS conducted in Japan only, lacking the concept of independent reviews by ethics committees and obtaining informed consent, which are important elements of clinical studies. Although the drug re-evaluation system in Japan works well, it does not cover all issues of the post-marketing phase.

In this study, we focused on attitudes toward obtaining informed consent for the collection and provision of data beyond the scope of routine clinical practice with respect to various issues in research ethics. As shown in Table 4, 42.7% of respondents selected “collect and provide data beyond clinical routine practice separate to PMS with informed consent”. In addition, although the use of questionnaire surveys was accepted by almost half of respondents, other procedures, namely, extra blood sampling, provision of images, monitoring and controlled studies, were supported by only 30% or less. Beyond practical factors such as workload, attention should be given to establishing an ethics infrastructure and global harmonization of PMS with regard to the Japanese PMS system.

This study evaluated physicians’ views and attitudes toward PMS from a practical and ethical viewpoint. We found that many physicians consider PMS to be important but burdensome. From the viewpoint of research ethics, many physicians considered it improper within the present PMS framework to collect and provide data beyond the scope of routine clinical practice without obtaining informed consent. However, given the limitations of this single-institution study, further research should be conducted to collect data for developing a suitable infrastructure for PMS.
